# Machine learning-aided analyses of thousands of draft genomes reveal specific features of activated sludge processes

**DOI:** 10.1186/s40168-020-0794-3

**Published:** 2020-02-11

**Authors:** Lin Ye, Ran Mei, Wen-Tso Liu, Hongqiang Ren, Xu-Xiang Zhang

**Affiliations:** 1 State Key Laboratory of Pollution Control and Resource Reuse, School of the Environment, Nanjing University, Nanjing, Jiangsu China; 2 Department of Civil and Environmental Engineering, University of Illinois at Urbana-Champaign, Urbana, IL USA

**Keywords:** Activated sludge, Metagenomics, Machine learning

## Abstract

**Background:**

Microorganisms in activated sludge (AS) play key roles in the wastewater treatment processes. However, their ecological behaviors and differences from microorganisms in other environments have mainly been studied using the 16S rRNA gene that may not truly represent in situ functions.

**Results:**

Here, we present 2045 archaeal and bacterial metagenome-assembled genomes (MAGs) recovered from 1.35 Tb of metagenomic data generated from 114 AS samples of 23 full-scale wastewater treatment plants (WWTPs). We found that the AS MAGs have obvious plant-specific features and that few proteins are shared by different WWTPs, especially for WWTPs located in geographically distant areas. Further, we developed a novel machine learning approach that can distinguish between AS MAGs and MAGs from other environments based on the clusters of orthologous groups of proteins with an accuracy of 96%. With the aid of machine learning, we also identified some functional features (e.g., functions related to aerobic metabolism, nutrient sensing/acquisition, and biofilm formation) that are likely vital for AS bacteria to adapt themselves in wastewater treatment bioreactors.

**Conclusions:**

Our work reveals that, although the bacterial species in different municipal WWTPs could be different, they may have similar deterministic functional features that allow them to adapt to the AS systems. Also, we provide valuable genome resources and a novel approach for future investigation and better understanding of the microbiome of AS and other ecosystems.

**Video Abtract.**

## Background

Activated sludge (AS) is the largest biotechnology application in the world and is of eminent importance for the remediation of anthropogenic wastewater [1]. The pollutant removal functions of AS are achieved by microorganisms with diverse community structures, among which populations with important metabolic functions have been individually studied [2–4]. Meanwhile, AS is a unique engineered ecosystem that can be controlled by a variety of operating conditions, and its attributes make it attractive for microbial ecologists studying the behaviors of microbial community assembly [5, 6].

One major topic of AS microbiome research is investigating the core populations that are consistent occupants in a large number of AS communities and are potentially important contributors to the system performance. Such analysis has been performed using 16S rRNA gene sequencing at different scales, including one full-scale wastewater treatment plant (WWTP) in Hong Kong [7], 13 WWTPs in Denmark [8], 14 WWTPs in Asia and North America [9], and 269 WWTPs in 23 countries [1]. Core AS microbial communities were identified at both regional and global scales by counting shared species or operational taxonomic units (OTUs), implying that a small number of key microorganisms constitute an indispensable portion of the AS community regardless of geographical and operational variations. However, the 16S rRNA gene, despite a useful biomarker to explore microbial community and construct phylogeny, does not necessarily reflect microbial physiology [10]. Therefore, the in situ functions and ecological contributions of the identified core AS populations are still not clear. Moreover, vast metabolic diversity can be embedded in one species or OTU, which is usually defined at 97% sequence identity or even higher levels [11]. Thus, further investigation of the AS community is warranted using more advanced approaches that could resolve metabolic potentials with higher resolution.

Metagenomics aimed at recovering population genomes and annotating genetic potentials have been applied to AS and uncovered individual microorganisms or functions that are challenging to study using other methods [12–14], demonstrating that this approach is promising for revealing greater diversity at the functional level than the analysis of 16S rRNA gene sequences. However, few efforts have been made to resolve microbial ecology, such as the core-community phenomenon in AS, using metagenomics. Furthermore, metagenomics could facilitate a comparative analysis of microbiomes of AS and other ecosystems at functional level. Microorganisms associated with freshwater systems, soil, human feces, rainwater, and stormwater have been shown to seed the activated sludge via influent sewage [15, 16]. Comparing the populations in AS and various non-AS ecosystems could provide insights into how the AS microbial community is assembled and whether the AS populations possess unique functional features that are vital to the adaption to the conditions of wastewater treatment bioreactors.

The vast diversity observed in AS and tremendous information obtained by metagenomics present new data analysis challenges. Conventional approaches mainly rely on reducing dimensionality to retrieve and visualize ecological patterns. Ordination analyses such as nonmetric multidimensional scaling and principal coordinates analysis could only present the first two or three eigenvectors that account for a limited proportion of the entire variance. Phylogenetic analysis is based on one or multiple selected conserved genes out of thousands of genes in a prokaryotic genome, which inevitably results in loss of information. In recent years, machine learning approaches have received growing attention and have been applied in genomics research [17, 18]. Unlike conventional methods, they can automatically detect patterns in data with less expert handcrafting and are therefore suitable to handle and analyze large and complex datasets such as genomic and metagenomic data [18, 19]. They can further be used to disentangle the complexity and diversity in the AS community by comparing different AS systems and comparing AS with other environments.

Here, we present 2045 high- and medium-quality bacterial and archaeal metagenome-assembled genomes (MAGs) recovered from 114 global municipal AS samples, representing one of the largest assemblies of MAGs from the municipal AS microbiome. After the recovery of the vast genomic information, we aimed to address two questions. First, is there a significant core AS community at the MAG and protein level shared by a large number of WWTPs, or are there obvious plant-specific features in the AS MAGs? Second, are the AS MAGs similar to genomes of populations from other environments, or do they have unique environment-specific traits? In addition to a novel machine learning approach, a collection of conventional methods including genome and protein comparison, phylogenetics, and ordination was applied, and their results were compared.

## Results

### 2045 MAGs were obtained from AS of different WWTPs

**Fig. 1:**
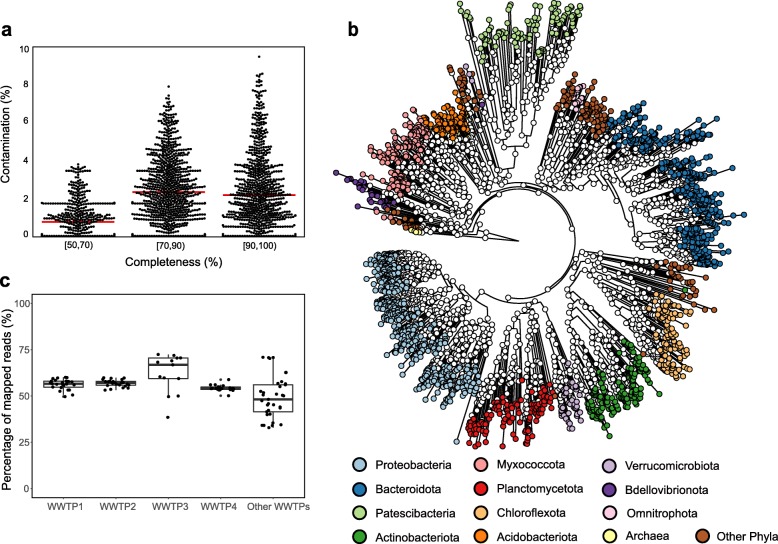
Overview of the 2045 MAGs assembled from 114 AS microbiomes. **a** Estimated completeness and contamination of the 2045 MAGs. The position of each horizontal red line refers to the mean contamination value of the corresponding group. **b** A maximum likelihood phylogenetic tree of the AS archaeal and bacterial MAGs based on universal core gene markers. The genome phylogenetic tree was generated using the universal PhyloPhlAn markers conserved across the bacterial and archaeal domains. A total of 98 MAGs with fewer than 80 universal markers were not included in this tree. The taxonomy of the MAGs was determined using GTDB-Tk, and it is shown in different colors. **c** Percentages of metagenomic sequencing reads of the different AS samples mapped to the 2045 MAGs

Approximately 1.35 Tb of metagenomic sequencing data generated from 114 AS samples of 23 municipal WWTPs located in eight countries were used to construct MAGs (Additional file 1: Figure S1, Table S1, Table S2). Among the 7548 bacterial and archaeal MAGs obtained, 2045 are estimated to have overall quality (defined as completeness − 5 × contamination) ≥ 50 [20]. The average completeness and contamination of the 2045 MAGs were 82.0% and 2.0%, respectively. Figure 1a shows that 743 of the 2045 MAGs are nearly complete (completeness ≥ 90%, average contamination 2.6%). The other two groups contain 845 (70% ≤ completeness < 90%) and 456 MAGs (50% ≤ completeness < 70%), and their average contamination values are 3.3% and 0.92%, respectively. The average contig number of these MAGs is 292, and the contig numbers have a moderate association with contamination level (Spearman’s rho = 0.47, *P* < 2.2e−16) but not with completeness level (Spearman’s rho = − 0.11, *P* = 4.3e−08) (Additional file 1: Figure S2). As shown in Additional file 1: Figure S2, most of the MAGs have good overall quality (high completeness and low contamination), while it was also found that some MAGs have relatively smaller contig numbers and medium-quality values (50–80%) (Additional file 1: Figure S2a), which leads to the relatively weak association between contig number and contamination level.

The 2045 MAGs were classified into 49 phyla (Fig. 1b and Additional file 1: Table S3). Among these MAGs, 21 were assigned to three archaeal phyla (*Halobacterota*, *Micrarchaeota*, and *Nanoarchaeota*). For bacteria, the phylum containing the highest number of MAGs was *Proteobacteria* (508 MAGs), followed by *Bacteroidota* (409 MAGs), *Patescibacteria* (178 MAGs), *Myxococcota* (164 MAGs), *Actinobacteriota* (161 MAGs), *Planctomycetota* (122 MAGs), *Chloroflexota* (114 MAGs), and *Acidobacteriota* (96 MAGs). The remaining MAGs were assigned to other miscellaneous bacterial phyla (Additional file 1: Table S3). To further understand the diversity among these MAGs, phylogenetic analysis was performed using the universal core gene markers predicted from each MAG [21]. Figure 1b shows that the clustering patterns in the tree are highly consistent with the taxonomy assignments, with *Proteobacteria* and *Bacteroidales* as the two most dominant clusters.

To estimate the representativeness of the MAGs for AS microbial genetic information, we mapped the metagenomic sequencing reads of each WWTP to the MAGs and calculated the percentage of mapped reads in each sample. As shown in Fig. 1c, 54–63% of reads (average per WWTP) of AS samples from the first four WWTPs, which have larger sequencing data volumes and significantly contribute to the AS MAG catalog, were mapped to the MAGs. For other WWTPs, the mapping ratios ranged from 34 to 72%.

### The AS MAGs show obvious plant-specific features

**Fig. 2:**
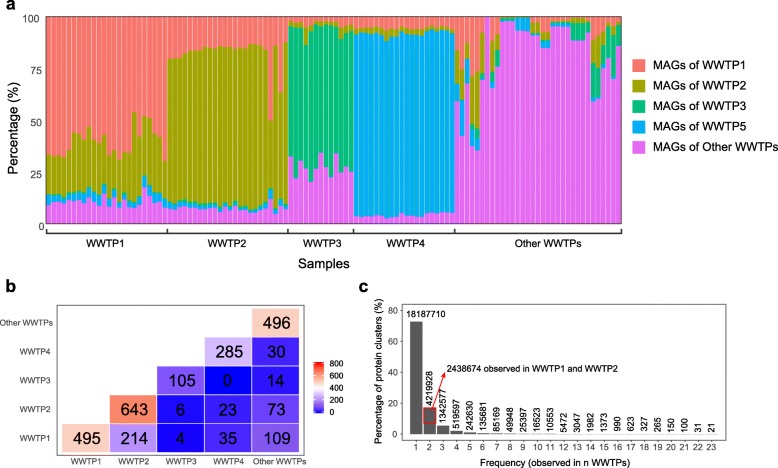
Comparison of MAGs and protein sequences in different WWTPs. **a** Relative abundance of metagenomic sequencing reads of each sample mapped to the MAGs from different WWTPs. **b** Numbers of MAG pairs with ANI > 95% between different WWTPs. The values on the diagonal also refer to the MAG number in each of the first four WWTPs and the total MAG number of other WWTPs. **c** Frequency distribution of protein clusters across WWTPs. The protein sequences predicted from all assembly contigs were clustered at an identity cutoff of 90% with CD-HIT, and then the protein clusters observed at each frequency were counted. The *y*-axis values were transformed to percentages, and the numbers on the top of bars refer to the absolute values of protein clusters observed in *n* WWTPs

To evaluate the plant-specific features of the MAGs, we first analyzed the distribution of reads mapped to the MAGs obtained from different plants. As shown in Fig. 2a, most (60–87%) of the mapped metagenomic reads from each WWTP were mapped to its own MAGs. A relatively small fraction of reads in each WWTP (approximately 33% in WWTP1, 32% in WWTP2, 35% in WWTP3, and 13% in WWTP4) were mapped to MAGs from other WWTPs. MAGs of WWTP1 and WWTPs shared more mapped reads than other WWTP pairs (approximately 20% of sequencing reads of WWTP1 and WWTP2 were mapped to each other’s MAGs), likely because they are located in the same city.

In addition to mapping reads to MAGs, we also computed the average nucleotide identity (ANI) values by comparing the MAGs with an all-against-all strategy. The results in Fig. 2b and Additional file 1: Figure S3 show that 214 MAG pairs have ANI > 95% between WWTP1 and WWTP2, suggesting that these 214 bacterial or archaeal species (43% MAGs in WWTP1 and 33% MAGs in WWTP2) were shared between WWTP1 and WWTP2. However, the numbers of potentially shared species between other WWTPs were relatively small. For example, no MAG pairs with ANI > 95% were observed between WWTP3 and WWTP4, and only four MAG pairs with ANI > 95% were found between WWTP1 and WWTP3. A number of MAG pairs were also observed between WWTP1 and “other WWTPs” (109) and between WWTP2 and “other WWTPs” (73). This is probably because a large fraction (9/19) of WWTPs in “other WWTPs” are located in China and near WWTP1 and WWTP2 (Additional file 1: Table S1).

Since the MAGs represent only part (34 to 72%) of the AS microbiome according to the mapping results, we also conducted a pairwise comparison of protein sequences predicted from all assembled contigs of the first four WWTPs. Other WWTPs were not included in this comparison because of their low sequencing depths. As shown in Additional file 1: Figure S4, 62% of proteins predicted from WWTP1 are highly similar (identity > 90%) to those of WWTP2. However, only a small number of proteins predicted from WWTP3 (10–27%) and WWTP4 (7.9–28%) have highly similar hits (identity > 90%) in other WWTPs. We further identified 24,850,093 clusters (identity cutoff 90%) from the 44,212,953 protein sequences predicted from all AS samples. A frequency distribution plot (Fig. 2c) shows that 73.2% of the protein clusters were found in one WWTP, and 17.0% were found in two WWTPs. Among the protein clusters observed in two WWTPs, over half (57.8%) were shared by WWTP1 and WWTP2, which were located in the same city. Only 0.1% of total protein clusters were present in > 10 WWTPs. The protein comparison results confirmed the results of read mapping and ANI calculation. It further suggested that, although a certain amount of proteins and MAGs may be shared by different WWTPs, a large proportion of bacterial populations in different WWTPs are largely different at both the DNA and protein levels, i.e., the bacterial genomes have plant-specific features.

### Phylogeny and functional features cannot well separate MAGs from AS and MAGs from other environments

**Fig. 3:**
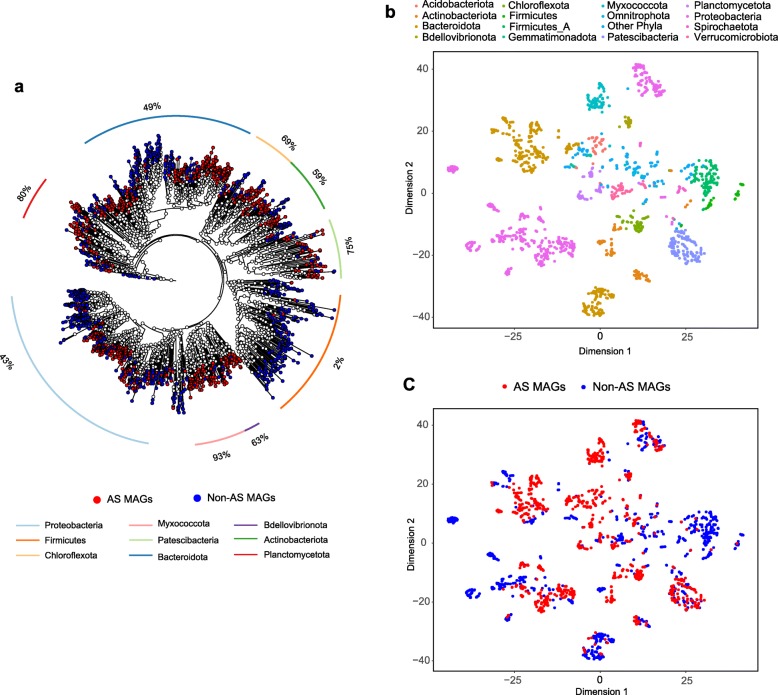
Phylogenetic and functional comparison of AS MAGs and non-AS MAGs. **a** A whole-genome maximum likelihood phylogenetic tree consisting of AS MAGs and non-AS MAGs. One thousand MAGs randomly selected from AS bacterial MAGs and 1000 MAGs randomly selected from other environments (Parks et al. [20]) were used to build this whole-genome tree with the same methods as in Fig. 1b. The outside percent value refers to the relative abundance of AS MAGs in each clade. **b** Clustering of the AS and non-AS MAGs based on the COG presence/absence matrix with the t-SNE algorithm. The 2000 MAGs in **a** were used to generate this figure. The points representing MAGs are colored according to the taxonomy of each MAG. **c** The same clustering plot as in **b**, with the red points representing AS MAGs and blue points representing non-AS MAGs

In addition to comparing MAGs among different WWTPs, we also explored whether the 2024 bacterial AS MAGs obtained in this study could be distinguished from the 7164 MAGs of other non-engineered (natural and animal/human-related) environments [20]. We constructed a maximum likelihood phylogenetic tree encompassing 1000 randomly selected AS MAGs and 1000 randomly selected non-AS MAGs (Fig. 3a). The tree shows that both AS and non-AS MAGs are distributed in a wide range of phyla. Non-AS MAGs were dominant in the *Firmicutes* clade (which contained only 2% AS MAGs). More AS MAGs than non-AS MAGs belonged to *Myxococcota* (93% AS MAGs) and *Planctomycetota* (80% AS MAGs). Considerable amounts of both AS and non-AS MAGs were present in most of the remaining clades. These patterns remained basically unchanged when the number of AS and non-AS MAGs used for tree construction increased. Overall, the large-scale phylogenetic analysis based on random selection shows that the AS MAGs are phylogenetically interspersed among non-AS MAGs, and no clear separation patterns were observed.

We further investigated the differences between AS and non-AS MAGs by annotating them with the database of clusters of orthologous groups of proteins (COGs). As proteins in each COG have the same domain architecture and likely have the same function [22], comparison of COG profiles may reflect the different functions encoded in the MAGs. A COG presence/absence matrix was generated for the 2024 bacterial AS MAGs and 7164 non-AS bacterial MAGs. A t-Distributed Stochastic Neighbor Embedding (t-SNE) analysis based on the COG presence/absence matrix was able to separate MAGs associated with different phyla (Fig. 3b). However, no clear grouping patterns between AS MAGs and non-AS MAGs (Fig. 3c) were observed, which was similar to the results of the phylogenetic tree. Most of the AS and non-AS MAGs were widely distributed and co-present in most phyla, except that few AS MAGs were observed in *Firmicutes* and some AS MAGs were separated from non-AS MAGs in the *Bacteroidota* cluster.

### A machine learning approach to distinguish between AS and non-AS MAGs based on COGs

**Fig. 4:**
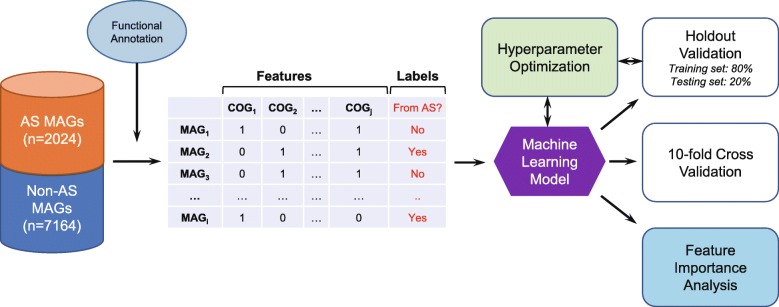
Flowchart of the implementation of machine learning for predicting AS and non-AS MAGs

**Fig. 5:**
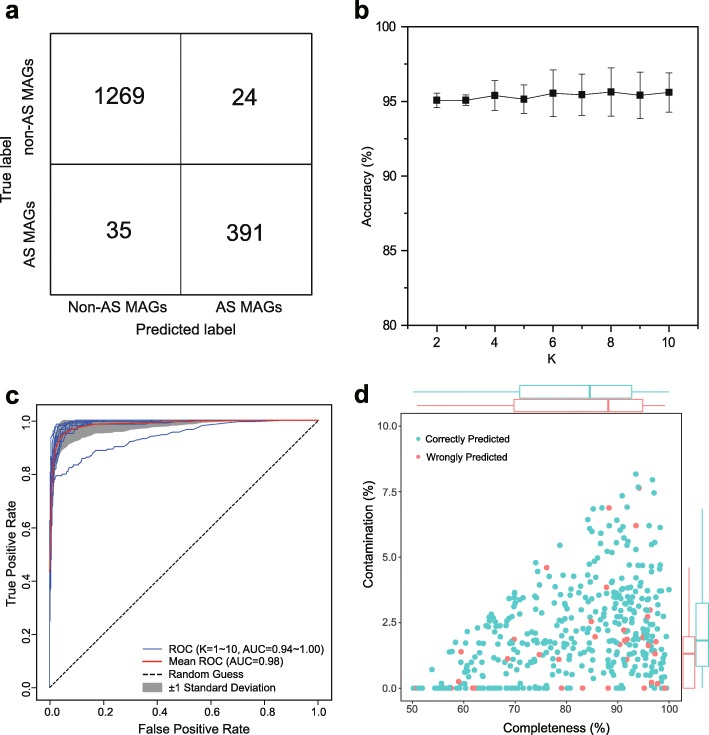
Performance of the random forest model. **a** Confusion matrix showing the performance of the random forest model on the 20% testing data group of the holdout validation. **b** Prediction accuracy of the random forest model determined based on 10-fold cross-validation. **c** ROC curves for evaluating the random forest model created from 10-fold cross-validation. **d** The completeness and contamination of correctly predicted MAGs and wrongly predicted MAGs. Boxplots along the *x*- and *y*-axes show the means and quartiles of the completeness and contamination values of correctly and wrongly predicted MAGs

We further explored whether machine learning can better distinguish between AS and non-AS MAGs. To do so, the COG presence/absence matrix generated from the 2024 AS and 7164 non-AS MAGs was used as an input of the random forest model (Fig. 4). After the model was constructed and trained, its accuracy and applicability were further evaluated. Both the holdout method and *k*-fold cross-validation were applied to verify the model to avoid the overfitting issue. For the holdout method, the dataset was divided into two partitions as testing (20%) and training (80%) sets. The number of trees is an important parameter affecting the accuracy of the random forest algorithm and should be tuned. As shown in Additional file 1: Figure S5, after the tree number (*n* estimators) was increased to 200, the accuracy did not increase with the number of trees, and other parameters (tree depth and max features) were also simultaneously optimized (Additional file 1: Figure S5). With optimized parameters (*n* estimators 300, tree depth 20, and max features 100), the training and testing data groups were analyzed (Fig. 5a), and the overall prediction accuracy of the random forest model achieved 96.6% (94% for AS and 97% for non-AS MAGs, Additional file 1: Table S4). Particularly, the recall (i.e., true positive rate) for non-AS MAGs was 98%, which was higher than that of the AS MAGs (91%). This result suggests that approximately 9% of AS MAGs were wrongly classified as non-AS MAGs. The F1-score, which is the harmonic average of the precision and recall, of AS and non-AS MAGs was 0.93 and 0.98, respectively. The classification accuracy obtained from 10-fold stratified cross-validation ranged from 95.0 to 95.6% (Fig. 5b), suggesting that the model is reliable and accurate, and no overfitting was observed. Receiver operating characteristic (ROC) curves also demonstrated the excellent performance (area under the ROC curve (AUC) ranged from 0.94 to 1; for the mean ROC curve, AUC = 0.98) of the random forest model (Fig. 5c).

We further investigated the quality (completeness and contamination) and phylogeny of the wrongly predicted MAGs. Figure 5d indicates that the wrongly predicted MAGs were evenly distributed among correctly predicted MAGs. There was no significant difference between the contamination values of the two groups of MAGs (*t* test, *P* < 0.05). The average contamination of the wrongly predicted MAGs (1.7%) was lower than that of the correctly predicted MAGs (2.2%), and the average completeness of the wrongly predicted MAGs (82.1%) was slightly higher than that of the correctly predicted MAGs (81.6%). This suggests that the overall quality of wrongly predicted MAGs is better than that of correctly predicted MAGs. Therefore, completeness and contamination levels may not be the major reasons leading to incorrect prediction. Phylogenetic analysis showed that erroneously predicted MAGs were distributed in various phyla, while many were associated with *Proteobacteria*, which was inherently diverse (Additional file 1: Figure S6).

### Different functional features between AS and non-AS MAGs

**Fig. 6:**
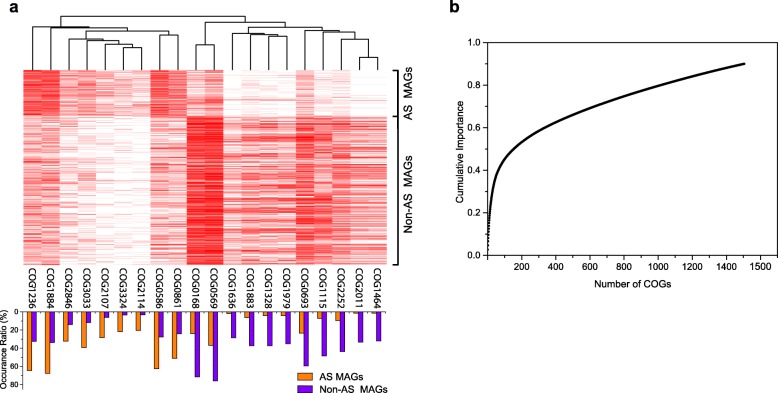
Feature importance determined by the random forest model. **a** The presence/absence of the top 20 COGs (with the highest importance values) in each MAG (heatmap). The “red” and “white” colors represent presence and absence, respectively. The bar plot shows the percentage of MAGs carrying each COG in the AS MAG group and in the non-AS MAG group. The importance values and descriptions are shown in Additional file 1: Table S5. **b** Cumulative importance values of the COGs

During the random forest model training, an importance value was assigned to each COG. The COGs with higher importance values were more informative when the model was used to predict whether a MAG was from AS. Therefore, by analyzing the importance of each COG, the functions that differentiate the sources of MAGs can be identified. Figure 6a shows the presence/absence of the top 20 COGs based on the importance value among the MAGs (see Additional file 1: Table S5 for the importance values and descriptions). Some COGs (e.g., COG1979, 1328, 1464, 2011, and 1636) were clearly rarely present in AS MAGs. Proteins of these COGs are related to anaerobic metabolisms or functions, such as alcohol dehydrogenase and anaerobic ribonucleoside-triphosphate reductase. In contrast, several COGs (e.g., COG3324, 2114, 2107, and 3303) were more frequently observed in AS MAGs than in MAGs from other environments. Proteins of COG3324 and COG 2114 are related to sensing the nutritional contents of the surrounding media or other environmental signals [23]. Proteins of COG 3033 were annotated as tryptophanase, which catalyzes the beta-elimination reaction of l-tryptophan to yield indole, ammonium, and pyruvate, and the produced indole molecules may affect biofilm formation and multidrug exporters [24].

Many COGs besides the top 20 also contributed to the machine learning-based prediction. Among them, 148 COGs accounted for 50% of the cumulative importance, and approximately 1500 COGs were needed to reach a cumulative importance of 90% (Fig. 6b). This result indicates the highly diverse functional features of the AS microbiomes and the strong capability of the machine learning approach in capturing complex information. It also explained why the conventional phylogenetic and ordination approaches failed to separate the AS and non-AS MAGs.

## Discussion

Despite the important roles of AS microorganisms in removing various pollutants from wastewater, the microbiome in AS remains largely uncharacterized. Based on metagenomic assembly and binning strategies, this study constructed an AS genome catalog consisting of 2024 bacterial and 21 archaeal MAGs recovered from 114 global municipal AS samples. This catalog likely represents the largest reported AS genome collection. Its coverage of the bacteria in AS systems is considered to be high, as up to 50–70% of the metagenomic sequencing reads could be mapped to the MAGs. Thus, this catalog could enable us to comprehensively profile the AS bacterial community structures and functions in a higher-resolution manner.

We found that the bacterial MAGs obtained from different WWTPs could be largely different according to DNA and protein comparisons, especially for WWTPs located in geographically distant areas. This suggests that AS MAGs may have plant-specific features at the genetic level and is consistent with a recent study based on 16S rRNA gene sequencing showing that municipal AS has a small, global core bacterial community [1]. Since MAGs contain much more genetic information and have more variants than 16S rRNA genes, it can be inferred that the genomes of the bacteria within the small core determined based on the 16S rRNA gene could also be largely different in different WWTPs. Therefore, the number of highly similar bacterial genomes present in different WWTPs might be very limited. The observation of small-core populations is in line with the previously reported functional redundancy in AS ecosystems [25, 26]. Although the overall functions of AS in all municipal WWTPs are carbon and nutrient removal, different operational parameters and wastewater compositions may lead to significantly different microbial communities with similar functions in different WWTPs. Moreover, we found that the similarity between MAGs of WWTP1 and WWTP2 located in the same city is higher than the similarity between MAGs of other WWTPs (Fig. 2 and Additional file 1: Figure S4). This is probably due to the similar wastewater compositions and environmental conditions in WWTP1 and WWTP2. This finding agrees with previous reports [8, 9] that regional WWTPs have more core bacteria taxa than global WWTPs [1]. Overall, the low similarity of the MAGs and proteins between different WWTPs suggests that extremely high genetic diversity is present in the AS ecosystem.

Due to the extremely high genetic complexity in AS, the phylogenetic tree and COG ordination analysis failed to distinguish between AS MAGs and non-AS MAGs. The major reason is that phylogenetic analysis and COG ordination are processes developed to reduce the dimensionality of multivariate data. For phylogenetic tree construction, only a limited number, usually a few hundreds, of genes coding universally conserved proteins are selected among 2000–3000 genes in a bacterial genome [21], leading to a concomitant loss of genetic information. Further loss occurs when the sequencing data are converted into distances (distance-matrix methods) or likelihood estimations (maximum likelihood methods) or when singular sites are discarded (parsimony methods) [27, 28]. The ordination methods (including t-SNE) also suffer from information loss due to the dimension reduction [29]. Although dimension reduction is important in some cases to summarize significant information from redundant high-dimensional data [30], its application could miss the subtle dependencies in the datasets; for instance, the differences between AS and non-AS MAGs were not captured in this study. Here, we found that a machine learning approach (random forest model) accurately distinguished between AS MAGs and non-AS MAGs based on COG presence/absence because the random forest algorithm could take full advantages of high-dimensional data by constructing a multitude of decision trees [31].

The high prediction accuracy of machine learning also suggests that municipal WWTPs can select bacteria with specific functions. Although the bacterial species in different municipal WWTPs could be different [32], they may have similar deterministic functional traits to adapt themselves to the AS system. This idea complements the recent finding that the stochastic process is more important than deterministic factors in shaping the community assembly in AS based on 16S rRNA gene sequencing [1]. The higher resolution of genome-level analysis reveals that AS bacterial genomes have specific functional traits despite stochastic community assembly. Based on the random forest algorithm, we identified several functional features that are likely important for the bacteria in AS systems. Some features are primarily related to aerobic conditions in municipal WWTP bioreactors. Besides, we also found that COGs involved in sensing the nutritional contents or other environmental signals are important for bacteria in AS. This is probably related to the more frequent changes of loading rate and other conditions in wastewater treatment bioreactors than other natural environments (e.g., soil and sea water). Another functional feature is regulating of the biofilm formation, which also important for AS because most bacteria in AS are involved in floc (a specialized type of biofilm) formation. However, the role of many other COGs and their co-occurrence contributions to the machine learning model remain unexplained. It should also be noted that the protein functions inferred based on COG annotation may not be sufficient to reflect the detailed functional features of the AS. Future efforts are needed to investigate and confirm the functions of the proteins assigned to these COGs.

Despite the high prediction accuracy of the random forest algorithm, we also noted some false positive and false negative predictions. Further analysis shows that these erroneous results were not due to the quality (completeness and contamination) of the MAGs, suggesting that the random forest model could handle datasets with missing values (incomplete MAGs) and a certain level of noise (contaminated MAGs) well [33]. A small number of erroneous results are reasonable because AS is an open ecosystem, and extraneous microorganisms could be introduced into the AS through incoming raw sewage [8] or upstream biological processes [34]. In addition, the microorganisms in AS could also be easily spread to other environments via effluent discharge to receiving water bodies [35]. These stochastic propagation processes could not be captured by the machine learning model, and other technologies should be applied to identify these minor species.

Although high percentages of the metagenomic sequencing reads (50–75% for most samples) were included in the AS MAGs obtained in this study, a large number of bacterial genomes in the AS still remain unavailable due to the high complexity of the AS microbiome and microdiversity issues, which significantly hampers genome assembly and binning [12, 36]. Also, many MAGs may not be obtained due to the relatively low sequencing depths of some samples analyzed in this study (Additional file 1: Table S1). We anticipate that these genomes also possess functional features similar to those of the MAGs obtained in this study, and future investigations with higher sequencing depth based on long-read sequencing [37] or single-cell sequencing [38] are needed to confirm this hypothesis. In addition, although thousands of COGs were identified by the machine learning model as important functional features to distinguish between AS MAGs and non-AS MAGs, most of them could not be well annotated. Further investigation of these proteins would be beneficial to improve our understanding of the microbial ecology of AS systems and provide a theoretical foundation for optimizing AS processes. Moreover, it should be noted, like other metagenomic studies, incorrect contig assembly and false assignment of assembled contigs to MAGs [39] may also occur in the MAG catalog of this study. Therefore, caution should be taken when using this dataset in future studies and various analyses and experiments are encouraged to confirm the results.

## Conclusions

In summary, our work provides one of the largest genome resources for investigation of the AS microbiome. Based on this, we found that the AS MAGs have obvious plant-specific features and that few genomes and proteins are shared by different WWTPs, especially for WWTPs located in geographically distant areas. Despite the differences, specific functional traits of AS MAGs, including functions related to aerobic metabolism, nutrient sensing/acquisition, and biofilm formation, were identified by a machine learning approach based on the COG presence/absence matrix. These features are likely important for bacteria to adapt themselves in AS systems. By applying the machine learning approach, AS MAGs could be differentiated from non-AS MAGs with an accuracy of 96.6%. The results demonstrated that machine learning approach could be a powerful tool for understanding the microbial ecology in different ecosystems.

## Methods

### Activated sludge sampling

In this study, 57 AS samples were collected from the aeration tanks of 11 full-scale municipal WWTPs in 8 cities of China for metagenomic sequencing (Additional file 1: Table S1). For the two WWTPs in Nanjing City, time-series sampling was conducted every month from January 2014 to December 2015, and 24 samples were obtained from each of the two WWTPs. For other WWTPs, sampling was conducted only once in each plant during the period from April 2017 to July 2017. Detailed information about the WWTPs is shown in Additional file 1: Table S1. All sludge samples were fixed in 50% (v/v) ethanol aqueous solution and transported on ice to the laboratory for DNA extraction.

### DNA extraction and metagenomic sequencing

DNA was extracted from the AS samples using the FastDNA™ SPIN Kit for Soil (MP Biomedicals, Irvine, CA, USA) following the manufacturer’s protocol. The DNA concentration and quality were determined using a NanoDrop One spectrophotometer (Thermo Fisher Scientific, Waltham, MA, USA) and agarose gel (2%) electrophoresis. Metagenomic sequencing was conducted to obtain the entire genomic information from the sludge samples. DNA extracted from each AS sample was used for metagenomic library construction and then sequenced on an Illumina HiSeq X Ten platform (San Diego, CA, USA) with a paired-end (2 × 150) sequencing strategy. The raw metagenome reads have been deposited in the NCBI Sequence Read Archive and are available under the BioProject PRJNA556302.

### Collection of public activated sludge metagenomic data and metagenome-assembled genomes

In addition to the 57 AS metagenomes sequenced in this study, we also downloaded 57 other municipal AS metagenomic datasets reported in previous studies for assembly and genome binning. All of the datasets were generated on the Illumina HiSeq platform with paired-end sequencing strategy. The accession numbers and information of these datasets are shown in Additional file 1: Table S1, Table S2, and Fig. S1.

Moreover, a few thousands of bacterial MAGs in a previous study [20] were also used in this study. The MAGs obtained from the anaerobic digesters and laboratory-scale wastewater treatment reactors in this catalog were excluded. Because the seed sludge of these reactors is usually activated sludge, but the influent and operational conditions may be quite different from those of the typical aerobic reactors in municipal wastewater treatment plants. Therefore, their microbial communities may be quite different from those of the typical activated sludge. Finally, 7164 bacterial draft genomes recovered from the metagenomes of different environments in the previous study [20] were used to prepare the input data for the machine learning model.

### Metagenomic assembly and contig binning

The metagenomic data were trimmed and quality-filtered using Trimmomatic v 0.32 [40] with default parameters. Then, clean reads from all samples of each WWTP were assembled into contigs using MEGAHIT v1.1.1 [41] with the following parameters: --k-min 41 --min-contig-len 1000. Then, the clean reads of each sample were mapped to the assembled contigs using Bowtie2 v 2.2.9 [42]. A depth file was generated with the jgi_summarize_bam_contig_depths included in MetaBAT2 [43] based on the mapping results. Then, draft genomes were recovered based on tetranucleotide frequency and contig abundance using MetaBAT2 v 2.12.1 [43]. The quality of the recovered genome bins was assessed by using CheckM v 1.0.7 [44]. Open reading frames were predicted in the assembled scaffolds using Prodigal v 2.6.1 [45], CD-HIT v 4.7 [46] was used to group protein sequences into clusters based on sequence identity, and Diamond v0.9.24.125 [47] was used to compare the protein sequences obtained from different WWTPs.

### Merging of compatible bins and genome refining

The “merge” command of CheckM v 1.0.7 [44] was used to identify pairs of bins that could be merged according to the following criteria: (1) the completeness increased by ≥ 10% and the contamination increased by ≤ 1% when the bin pairs were merged; (2) the differences between mean GC of the bins were within 3%; (3) the mean coverage of the bins had an absolute percentage difference ≤ 25%; and (4) the bins had identical taxonomic classifications as determined by CheckM.

Genome refining was conducted with RefineM v0.0.24 [20]. Briefly, contigs with a GC or tetranucleotide distance outside the 98th percentile of the expected distributions were identified and removed. Contigs were also removed if their mean coverage had an absolute percentage difference ≥ 50% when compared with the mean coverage of the bin. The “taxon_profile” command of RefineM was used to taxonomically classify the genes constituting each bin, and contigs with divergent taxonomic classifications were removed with the “taxon_filter” command of RefineM. In addition, contigs with 16S rRNA genes that appear incongruent with the taxonomic identity of each bin were also identified and removed with RefineM. Only MAGs with an overall quality ≥ 50 (defined as completeness −5 × contamination) were used for downstream analysis. After genome refining, the genome taxonomy was assigned using GTDB-Tk v 0.2.1 (https://github.com/Ecogenomics/GTDBTk). The ANIs between MAGs were determined using FastANI [48].

### Genome phylogenetic tree construction

The phylogenetic analyses were conducted with PhyloPhlAn [21] using the “dev” branch of the repository (https://bitbucket.org/nsegata/phylophlan/overview). The genome maximum likelihood phylogenetic tree was generated in Newick format using the 400 universal PhyloPhlAn markers conserved across the bacterial and archaeal domains with the following options: “--diversity high --accurate --min_num_markers 80.” To avoid the crowd of tree branches, we used 1000 randomly selected AS MAGs and 1000 randomly selected non-AS MAGs to construct the tree. The final tree was reconstructed for visualization using GraPhlAn v1.1.3 [49].

### Functional genomic analysis

To identify protein domains in a genome, we annotated all of the MAGs using Prokka v 1.13.3 [50] with default parameters, and all protein domains were classified in different COGs. Then, a COG matrix was derived with MAGs in rows and the presence/absence of the COGs in each MAG as columns:

$${\displaystyle \begin{array}{ccccc}\ & {\mathrm{COG}}_1& {\mathrm{COG}}_2& \dots & {\mathrm{COG}}_{\mathrm{j}}\\ {}{\mathrm{MAG}}_1& 0& 1& \dots & 1\\ {}{\mathrm{MAG}}_2& 1& 0& \dots & 0\\ {}\dots & \dots & \dots & \dots & \dots \\ {}{\mathrm{MAG}}_i& 0& 0& \dots & {n}_{ij}\end{array}}$$

where the matrix element *n*_*ij*_ equals 1 if MAG_*i*_ encodes a protein ortholog belonging to COG_*j*_ and equals 0 otherwise.

The COG matrix was used to perform t-SNE analysis with the Rtsne package (https://cran.r-project.org/web/packages/Rtsne) and was also used for the construction of the machine learning model.

### Development of the machine learning model

The COG matrix constructed based on the functional annotation of the MAGs obtained in the present study and the previous study [20] was used to formulate the machine learning model to distinguish bacteria from municipal AS and those from other environments. The final dataset consists of 9288 MAGs (2024 from AS and 7164 from other environments) and 2580 COGs and was used to train and test two machine learning models based on support vector machine and random forest algorithms. Random forest was chosen because it has higher accuracy than support vector machine. Moreover, the random forest algorithm is suitable for datasets with many features, especially when each of the features contributes little information [31].

The model training and evaluation were performed with scikit-learn (https://scikit-learn.org/), a Python package for machine learning. Both the holdout method and *k*-fold cross-validation were applied to verify the model. For the holdout method, the dataset was divided into two partitions as training (80%) and testing (20%) sets. The training set was used to train the model, and the unseen testing data were used to test the predictive ability. Overfitting is a common issue in machine learning that can occur in most models [51]. In this study, out-of-bag (OOB) estimates were applied to avoid overfitting. In addition, 10-fold cross-validation was conducted to verify that the model was not overfitted. The dataset was randomly partitioned into 10 mutually exclusive and approximately equal subsets, and one set was kept for testing while the others were used for training. This process was iterated with the 10 subsets. Furthermore, the COGs significantly contributing to the machine learning-based prediction were analyzed based on the feature importance provided by the random forest model.

## Supplementary information

**Additional file 1: Table S1.** Information about the WWTPs and activate sludge samples analyzed in this study. **Table S2.** Accession numbers of the metagenomic datasets used in this study. **Table S3.** Abundance of AS MAGs assigned to each phylum. **Table S4.** Prediction report of the random forest model. **Table S5.** Importance values and descriptions of the top 20 COGs identified by random forest model to differentiate the AS and non-AS MAGs. **Figure S1.** Geographical locations of the WWTPs where activated sludge samples were collected by us and other researchers. **Figure S2.** Associations between MAG completeness and number of contigs (a), and associations between MAG completeness and number of contigs (b). **Figure S3.** Venn diagram showing the shared and unique MAGs of WWTP1, WWTP2, WWTP3 and WWTP4. **Figure S4.** Profile of protein sequences identity between different WWTPs. The protein sequences predicted from all assembly contigs of each WWTP were compared each other with Diamond and then the best hits of the protein sequences were counted and summarized. **Figure S5.** Random forest parameter tuning and optimization. (a) Number of trees (n_estimators); (b) Tree depth; (c) Maximum features. **Figure S6.** Phylogeny of the erroneously predicted MAGs. The topology of this tree is exactly same with Fig. 1b. Extended lines were added to show positions of the erroneously predicted MAGs.

## Data Availability

The raw reads of AS metagenomes sequenced in this study have been deposited in the NCBI Sequence Read Archive and are available under the BioProject PRJNA556302. All sequence data analyzed in this study are available in public databases with the accession codes given in Additional file 1: Table S2. The MAGs obtained in this study are available in Figshare at https://figshare.com/projects/AS_MAGs/66554.
