# Supplementary material for: Machine learning-aided analyses of thousands of draft genomes reveal specific features of activated sludge processes
Source: Microbiome. 2020 Feb 11;8:16. doi: 10.1186/s40168-020-0794-3 (PMC7014675; doi:10.1186/s40168-020-0794-3)
Supplement: Supplementary file 2 — Additional file 1: Table S1. Information about the WWTPs and activate sludge samples analyzed in this study. Table S2. Accession numbers of the metagenomic datasets used in this study. Table S3. Abundance of AS MAGs assigned to each phylum. Table S4. Prediction report of the random forest model. Table S5. Importance values and descriptions of the top 20 COGs identified by random forest model to differentiate the AS and non-AS MAGs. Figure S1. Geographical locations of the WWTPs where activated sludge samples were collected by us and other researchers. Figure S2. Associations between MAG completeness and number of contigs (a), and associations between MAG completeness and number of contigs (b). Figure S3. Venn diagram showing the shared and unique MAGs of WWTP1, WWTP2, WWTP3 and WWTP4. Figure S4. Profile of protein sequences identity between different WWTPs. The protein sequences predicted from all assembly contigs of each WWTP were compared each other with Diamond and then the best hits of the protein sequences were counted and summarized. Figure S5. Random forest parameter tuning and optimization. (a) Number of trees (n_estimators); (b) Tree depth; (c) Maximum features. Figure S6. Phylogeny of the erroneously predicted MAGs. The topology of this tree is exactly same with Fig. 1b. Extended lines were added to show positions of the erroneously predicted MAGs. [file 40168_2020_794_MOESM1_ESM.docx]

Additional file

**Machine learning-aided analyses of thousands of draft genomes reveal specific features of activated sludge processes**

Lin Ye^1*^, Ran Mei^2^, Wen-Tso Liu^2^, Hongqiang Ren^1^, Xu-Xiang Zhang^1*^

^1^State Key Laboratory of Pollution Control and Resource Reuse, School of the Environment, Nanjing University, Nanjing, Jiangsu, China

^2^Department of Civil and Environmental Engineering, University of Illinois at Urbana-Champaign, Urbana, Illinois, USA

*Correspondence to:

Lin Ye (linye@nju.edu.cn)

Xu-Xiang Zhang (zhangxx@nju.edu.cn)

**Table S1**. Information about the WWTPs and activate sludge samples analyzed in this study

| **No.** | **WWTP ID** | **WWTP Name/Label** | **Process** | **Location** | **Sample Number** | **Data Size (G bps)** | **Reference** |
| --- | --- | --- | --- | --- | --- | --- | --- |
| 1 | WWTP1 | JXZ | A/A/O | Nanjing, China | 24 | 267.1 | This study |
| 2 | WWTP2 | DC | OD | Nanjing, China | 24 | 331.1 | This study |
| 3 | WWTP3 | Aalborg West | NA | Aalborg, Denmark | 13 | 274.7 | ([Munck et al. 2015](#_ENREF_6)) |
| 4 | WWTP4 | Ulu Pandan | CAS+MBR | Singapore | 20 | 180.8 | ([Law et al. 2016](#_ENREF_4)) |
| 5 | WWTP5 | QS | A/A/O | Zhuhai, China | 1 | 16.3 | This study |
| 6 | WWTP6 | HP | A/A/O | Guangzhou, China | 1 | 17.1 | This study |
| 7 | WWTP7 | JM | A/A/O | Xiamen, China | 1 | 18.0 | This study |
| 8 | WWTP8 | XC | A/A/O | Wuxi, China | 1 | 16.7 | This study |
| 9 | WWTP9 | DJQ | CAST | Wuxi, China | 1 | 16.7 | This study |
| 10 | WWTP10 | SZ1 | A/A/O | Suzhou, China | 1 | 19.6 | This study |
| 11 | WWTP11 | SZ2 | A/A/O | Suzhou, China | 1 | 18.2 | This study |
| 12 | WWTP12 | HBH | A-B process | Qingdao, China | 1 | 15.5 | This study |
| 13 | WWTP13 | SY | A/O | Shenyang, China | 1 | 15.3 | This study |
| 14 | WWTP14 | Manitowoc | NA | USA | 3 | 10.8 | ([Chu et al. 2018](#_ENREF_1)) |
| 15 | WWTP15 | Sheboygan | NA | USA | 3 | 9.1 | ([Chu et al. 2018](#_ENREF_1)) |
| 16 | WWTP16 | SwgA | CAS | Buenos Aires, Argentina | 4 | 14.4 | ([Ibarbalz et al. 2016](#_ENREF_2)) |
| 17 | WWTP17 | SwgB | CAS | Buenos Aires, Argentina | 4 | 18.1 | ([Ibarbalz et al. 2016](#_ENREF_2)) |
| 18 | WWTP18 | Kocejve | NA | Slovenia | 3 | 18.2 | ([McIlroy et al. 2016](#_ENREF_5)) |
| 19 | WWTP19 | AD | NA | Swiss | 1 | 5.7 | ([Ju et al. 2019](#_ENREF_3)) |
| 20 | WWTP20 | WT | NA | Swiss | 1 | 5.3 | ([Ju et al. 2019](#_ENREF_3)) |
| 21 | WWTP21 | TE | NA | Swiss | 1 | 6.1 | ([Ju et al. 2019](#_ENREF_3)) |
| 22 | WWTP22 | TU | NA | Swiss | 1 | 5.4 | ([Ju et al. 2019](#_ENREF_3)) |
| 23 | WWTP23 | EGA | NA | Demark | 3 | 52.0 | ([Munck et al. 2015](#_ENREF_6)) |

**Abbreviations in the “Process” column**: A/O, anoxic/aerobic; A/A/O, anaerobic/anoxic/aerobic; CAS, conventional activated sludge; CAST, cyclic activated sludge technology; OD: oxidation ditch; MBR, membrane bioreactor; NA, not available.

**Table S2**. Accession numbers of the metagenomic datasets used in this study

| **WWTP ID** | **Accession numbers** |
| --- | --- |
| WWTP1 | SRR9852202, SRR9852201, SRR9852204, SRR9852203, SRR9852206, SRR9852205, SRR9852208, SRR9852207, SRR9852210, SRR9852209, SRR9852186, SRR9852185, SRR9852184, SRR9852183, SRR9852190, SRR9852189, SRR9852188, SRR9852187, SRR9852178, SRR9852177, SRR9852213, SRR9852214, SRR9852211, SRR9852212 |
| WWTP2 | SRR9852217, SRR9852218, SRR9852215, SRR9852216, SRR9852219, SRR9852220, SRR9852176, SRR9852175, SRR9852181, SRR9852174, SRR9852180, SRR9852179, SRR9852182, SRR9852198, SRR9852200, SRR9852199, SRR9852164, SRR9852165, SRR9852166, SRR9852167, SRR9852168, SRR9852169, SRR9852170, SRR9852171 |
| WWTP3 | ERR712369, ERR712370, ERR712371, ERR712372, ERR712373, ERR712374, ERR712375, ERR712376, ERR712377, ERR712378, ERR712379, ERR712380, ERR712381, ERR712382, ERR712383, ERR712384 |
| WWTP4 | SRR3501849, SRR3501850, SRR3501851, SRR3501852, SRR3501853, SRR3501854, SRR3501855, SRR3501856, SRR3501857, SRR3501858, SRR3501859, SRR3501861, SRR3501862, SRR3501873, SRR3501884, SRR3501885, SRR3501886, RR3501887, RR3501888, SRR3501889 |
| WWTP5 | SRR9852172 |
| WWTP6 | SRR9852173 |
| WWTP7 | SRR9852197 |
| WWTP8 | SRR9852196 |
| WWTP9 | SRR9852195 |
| WWTP10 | SRR9852194 |
| WWTP11 | SRR9852193 |
| WWTP12 | SRR9852192 |
| WWTP13 | SRR9852191 |
| WWTP14 | SRR5570992, SRR5570991, SRR5571003 |
| WWTP15 | SRR5571009, SRR5571008, SRR5571011 |
| WWTP16 | SRR2107210, SRR2107211, SRR2107212, SRR2107213 |
| WWTP17 | SRR2107215, SRR2107216, SRR2107218, SRR2107219 |
| WWTP18 | ERR1076073, ERR1076074, ERR1076075 |
| WWTP19 | ERR2808645, ERR2808647 |
| WWTP20 | ERR2808646, ERR2808661 |
| WWTP21 | ERR2808648, ERR2808650 |
| WWTP22 | ERR2808649, ERR2808664 |
| WWTP23 | ERR712389, ERR712390, ERR712391 |

**Table S3**. Abundance of AS MAGs assigned to each phylum

| **Domain** | **Phylum** | **Number of bins** | **Relative abundance (%)** |
| --- | --- | --- | --- |
| Bacteria | Proteobacteria | 508 | 24.84 |
|  | Bacteroidota | 409 | 20.00 |
|  | Patescibacteria | 178 | 8.70 |
|  | Myxococcota | 164 | 8.02 |
|  | Actinobacteriota | 161 | 7.87 |
|  | Planctomycetota | 122 | 5.97 |
|  | Chloroflexota | 114 | 5.57 |
|  | Acidobacteriota | 96 | 4.69 |
|  | Verrucomicrobiota | 50 | 2.44 |
|  | Bdellovibrionota | 34 | 1.66 |
|  | Gemmatimonadota | 23 | 1.12 |
|  | Nitrospirota | 18 | 0.88 |
|  | Omnitrophota | 18 | 0.88 |
|  | Elusimicrobiota | 17 | 0.83 |
|  | Spirochaetota | 17 | 0.83 |
|  | Eisenbacteria | 13 | 0.64 |
|  | Armatimonadota | 11 | 0.54 |
|  | Cyanobacteriota | 10 | 0.49 |
|  | AABM5-125-24 | 6 | 0.29 |
|  | UBP1 | 5 | 0.24 |
|  | Firmicutes | 4 | 0.20 |
|  | Zixibacteria | 4 | 0.20 |
|  | Calditrichota | 4 | 0.20 |
|  | KSB1 | 4 | 0.20 |
|  | Campylobacterota | 3 | 0.15 |
|  | Firmicutes_A | 3 | 0.15 |
|  | Desulfobacterota | 3 | 0.15 |
|  | UBA10199 | 3 | 0.15 |
|  | OLB16 | 2 | 0.10 |
|  | Thermotogota | 2 | 0.10 |
|  | Margulisbacteria | 2 | 0.10 |
|  | BRC1 | 2 | 0.10 |
|  | Cloacimonadota | 2 | 0.10 |
|  | UBP10 | 2 | 0.10 |
|  | Nitrospirota_A | 2 | 0.10 |
|  | Synergistota | 1 | 0.05 |
|  | Fermentibacterota | 1 | 0.05 |
|  | Bipolaricaulota | 1 | 0.05 |
|  | Firmicutes_H | 1 | 0.05 |
|  | UBP7 | 1 | 0.05 |
|  | Fusobacteriota | 1 | 0.05 |
|  | Hydrogenedentota | 1 | 0.05 |
|  | Dependentiae | 1 | 0.05 |
| Archaea | Halobacterota | 5 | 0.24 |
|  | Micrarchaeota | 6 | 0.29 |
|  | Nanoarchaeota | 10 | 0.49 |

**Table S4**. Prediction report of the random forest model

| **Category** | **Precision** | **Recall** | **F1-score** |
| --- | --- | --- | --- |
| AS MAGs | 0.94 | 0.91 | 0.93 |
| Non-AS MAGs | 0.97 | 0.98 | 0.98 |

Precision=True Positive / (True Positive + False Positive)

Recall=True Positive / (True Positive + False Negative)

F1-score = 2*Precison*Recall / (Precison + Recall)

**Table S5**. Importance values and descriptions of the top 20 COGs identified by random forest model to differentiate the AS and non-AS MAGs

| COG | Importance | Description |
| --- | --- | --- |
| COG0168 | 0.035 | Trk-type K+ transport systems, membrane components |
| COG1115 | 0.025 | Na+/alanine symporter [Amino acid transport and metabolism] |
| COG0586 | 0.022 | Uncharacterized membrane protein DedA, SNARE-associated domain |
| COG0569 | 0.021 | Trk K+ transport system, NAD-binding component [Inorganic ion transport and metabolism] |
| COG2011 | 0.015 | ABC-type methionine transport system, permease component [Amino acid transport and metabolism] |
| COG3033 | 0.014 | Tryptophanase [Amino acid transport and metabolism] |
| COG1236 | 0.011 | RNA processing exonuclease, beta-lactamase fold, Cft2 family [Translation, ribosomal structure and biogenesis] |
| COG1328 | 0.010 | Anaerobic ribonucleoside-triphosphate reductase [Nucleotide transport and metabolism] |
| COG1464 | 0.010 | ABC-type metal ion transport system, periplasmic component/surface antigen [Inorganic ion transport and metabolism] |
| COG0693 | 0.009 | Putative intracellular protease/amidase [General function prediction only] |
| COG0861 | 0.009 | Membrane protein TerC, possibly involved in tellurium resistance [Inorganic ion transport and metabolism] |
| COG1636 | 0.008 | Predicted ATPase, Adenine nucleotide alpha hydrolases (AANH) superfamily [General function prediction only] |
| COG3324 | 0.007 | Predicted enzyme related to lactoylglutathione lyase [General function prediction only] |
| COG2107 | 0.007 | Menaquinone biosynthesis enzyme MqnD [Coenzyme transport and metabolism] |
| COG1979 | 0.007 | Alcohol dehydrogenase YqhD, Fe-dependent ADH family [Energy production and conversion] |
| COG1883 | 0.007 | Na+-transporting methylmalonyl-CoA/oxaloacetate decarboxylase, beta subunit [Energy production and conversion] |
| COG2252 | 0.006 | Xanthine/uracil/vitamin C permease, AzgA family [Nucleotide transport and metabolism] |
| COG2114 | 0.006 | Adenylate cyclase, class 3 [Signal transduction mechanisms] |
| COG2846 | 0.006 | Iron-sulfur cluster repair protein YtfE, RIC family, contains ScdAN and hemerythrin domains [Posttranslational modification, protein turnover, chaperones] |
| COG1884 | 0.006 | Methylmalonyl-CoA mutase, N-terminal domain/subunit [Lipid transport and metabolism] |


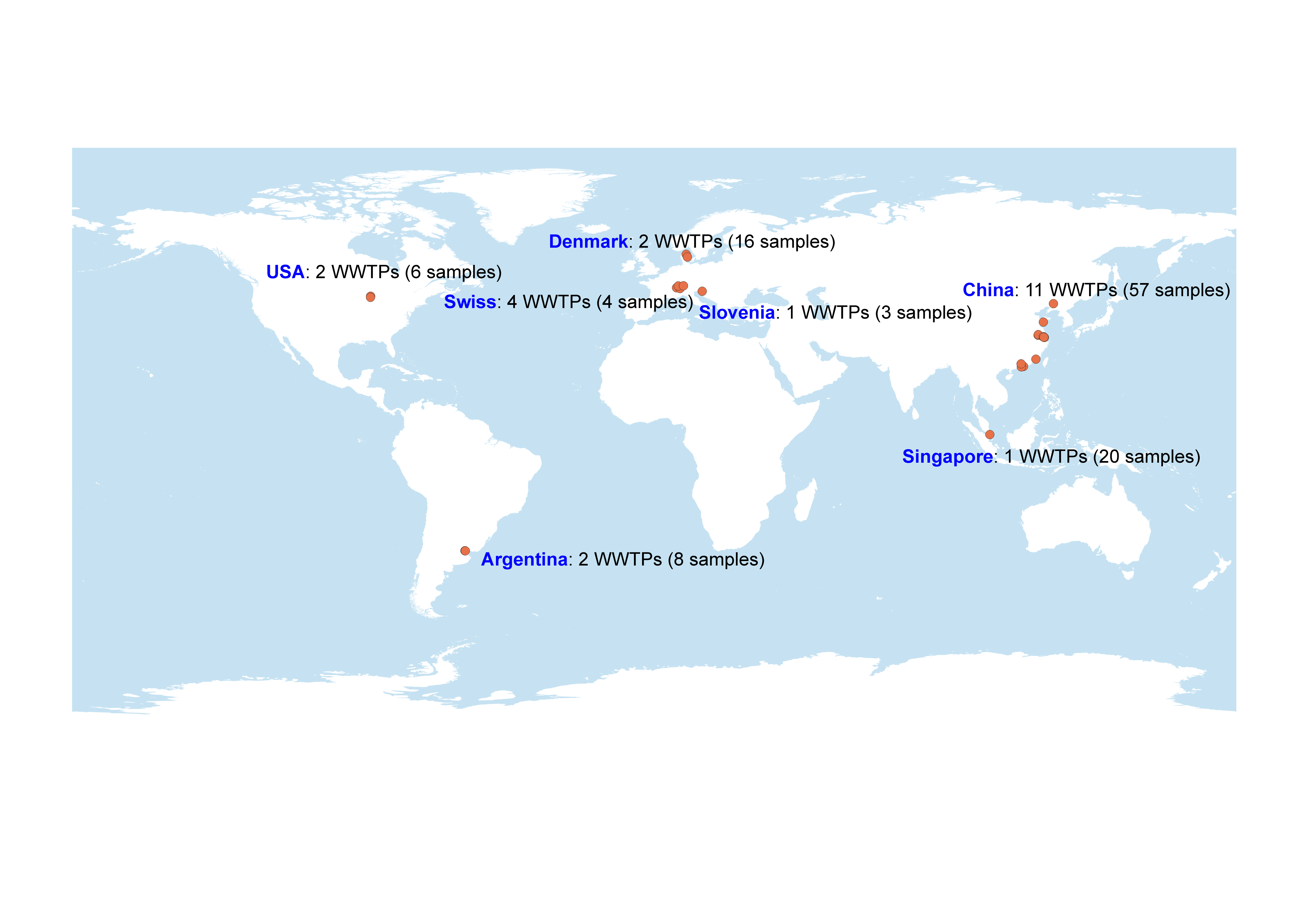


**Fig. S1** Geographical locations of the WWTPs where activated sludge samples were collected by us and other researchers.


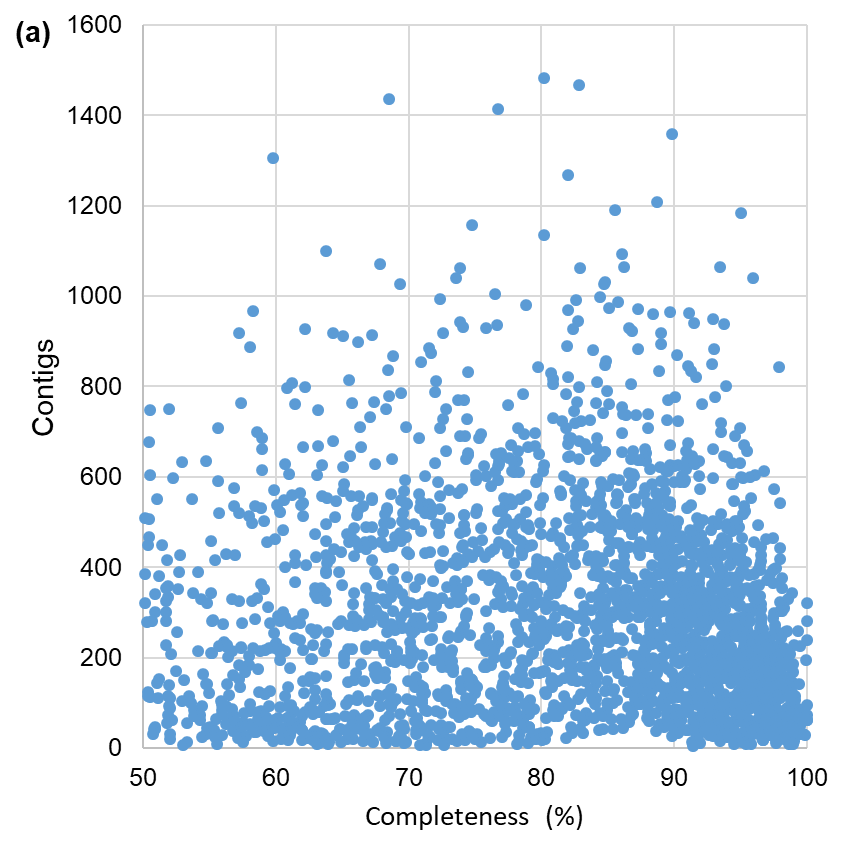

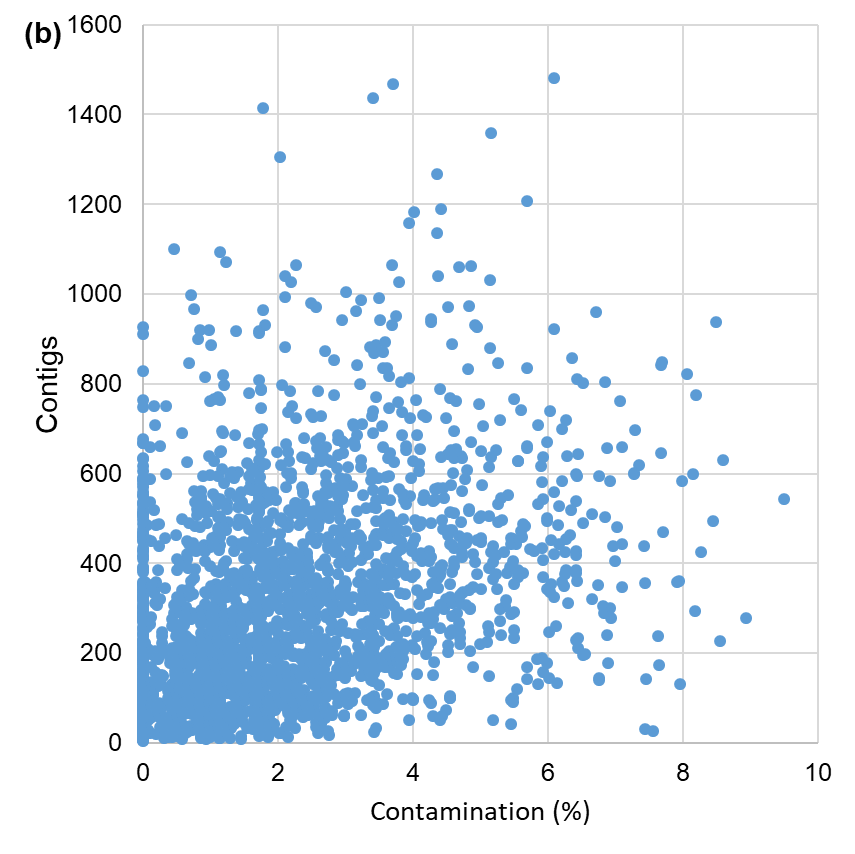


**Fig. S2** Associations between MAG completeness and number of contigs (a), and associations between MAG completeness and number of contigs (b).


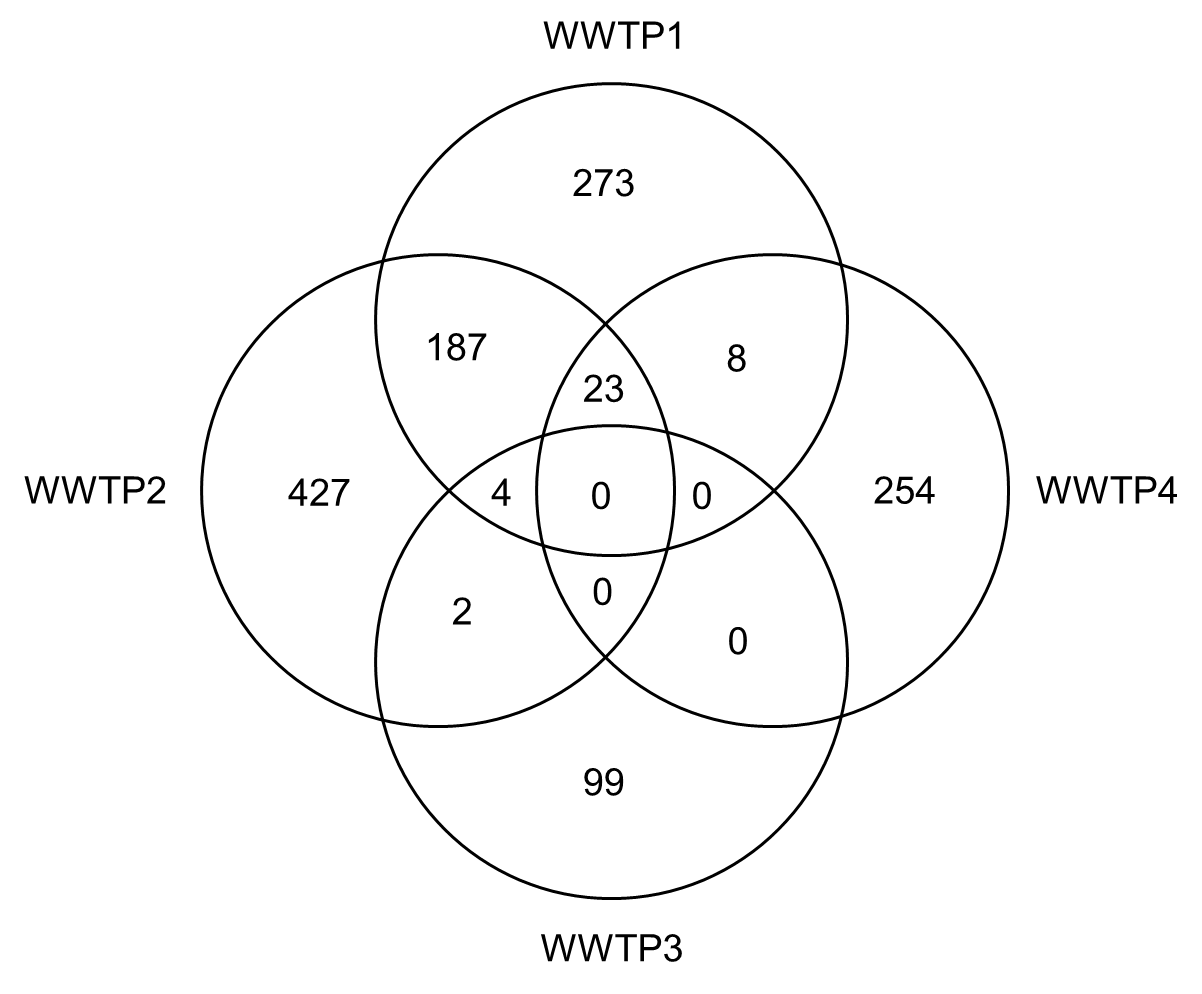


**Fig. S3** Venn diagram showing the shared and unique MAGs of WWTP1, WWTP2, WWTP3 and WWTP4.


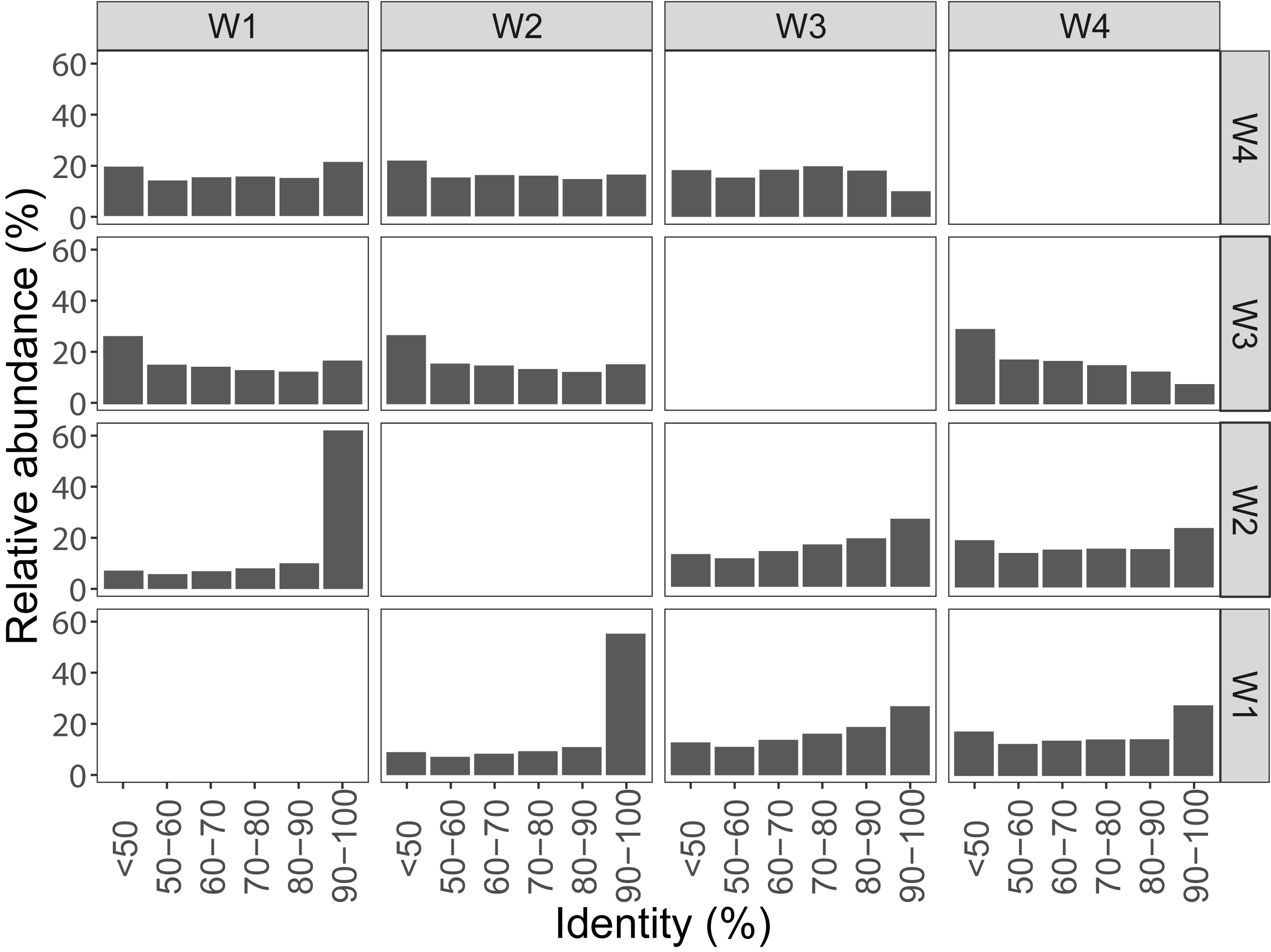


**Fig. S4** Profile of protein sequences identity between different WWTPs. The protein sequences predicted from all assembly contigs of each WWTP were compared each other with Diamond and then the best hits of the protein sequences were counted and summarized.


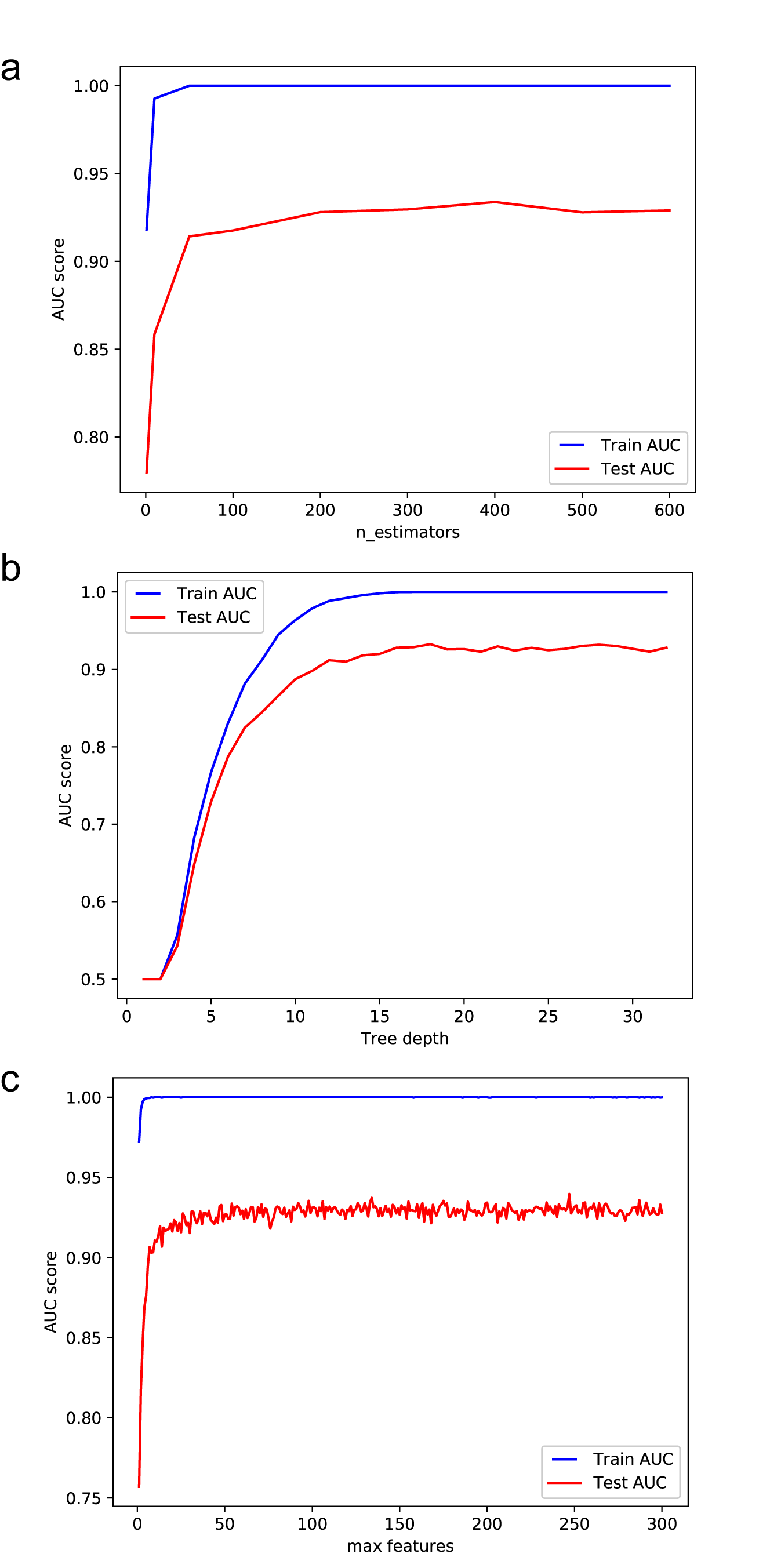


**Fig. S5** Random forest parameter tuning and optimization. (a) Number of trees (n_estimators); (b) Tree depth; (c) Maximum features.


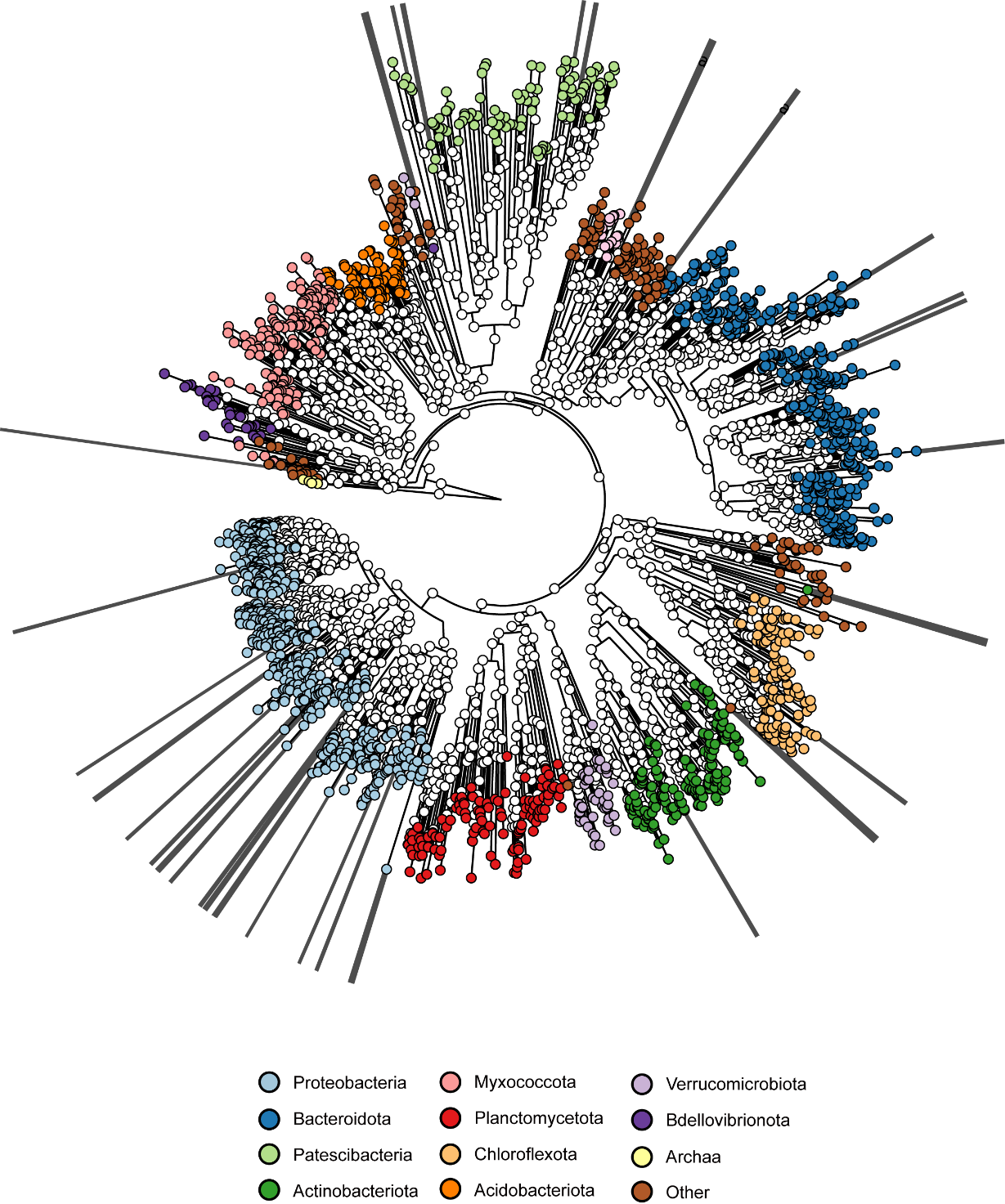


**Fig. S6** Phylogeny of the erroneously predicted MAGs. The topology of this tree is exactly same with Figure 1b. Extended lines were added to show positions of the erroneously predicted MAGs.

**References:**

Chu, B.T., Petrovich, M.L., Chaudhary, A., Wright, D., Murphy, B., Wells, G. and Poretsky, R., 2018. Metagenomics reveals the impact of wastewater treatment plants on the dispersal of microorganisms and genes in aquatic sediments. Applied and Environmental Microbiology 84(5), e02168-02117.

Ibarbalz, F.M., Orellana, E., Figuerola, E.L. and Erijman, L., 2016. Shotgun metagenomic profiles have a high capacity to discriminate samples of activated sludge according to wastewater type. Applied and Environmental Microbiology 82(17), 5186-5196.

Ju, F., Beck, K., Yin, X., Maccagnan, A., McArdell, C.S., Singer, H.P., Johnson, D.R., Zhang, T. and Bürgmann, H., 2019. Wastewater treatment plant resistomes are shaped by bacterial composition, genetic exchange, and upregulated expression in the effluent microbiomes. The ISME journal 13, 346-360.

Law, Y., Kirkegaard, R.H., Cokro, A.A., Liu, X., Arumugam, K., Xie, C., Stokholm-Bjerregaard, M., Drautz-Moses, D.I., Nielsen, P.H. and Wuertz, S., 2016. Integrative microbial community analysis reveals full-scale enhanced biological phosphorus removal under tropical conditions. Scientific reports 6, 25719.

McIlroy, S.J., Karst, S.M., Nierychlo, M., Dueholm, M.S., Albertsen, M., Kirkegaard, R.H., Seviour, R.J. and Nielsen, P.H., 2016. Genomic and in situ investigations of the novel uncultured chloroflexi associated with 0092 morphotype filamentous bulking in activated sludge. The ISME journal 10, 2223-2234.

Munck, C., Albertsen, M., Telke, A., Ellabaan, M., Nielsen, P.H. and Sommer, M.O., 2015. Limited dissemination of the wastewater treatment plant core resistome. Nature communications 6, 8452.
